# Results of Gamma Knife Radiosurgery in Acromegaly

**DOI:** 10.1155/2012/342034

**Published:** 2012-02-19

**Authors:** Alberto Franzin, Giorgio Spatola, Marco Losa, Piero Picozzi, Pietro Mortini

**Affiliations:** Department of Neurosurgery and Radiosurgery, Division of Neuroscience, IRCCS San Raffaele, 20132 Milan, Italy

## Abstract

*Objective*. Single-session radiosurgery with Gamma Knife (GK) may be a potential adjuvant treatment in acromegaly. We analyzed the safety and efficacy of GK in patients who had previously received maximal surgical debulking at our hospital. *Methods*. The study was a retrospective analysis of hormonal, radiological, and ophthalmologic data collected in a predefined protocol from 1994 to 2009. The mean age at treatment was 42.3 years (range 22–67 yy). 103 acromegalic patients participated in the study. The median follow-up was 71 months (IQ range 43–107). All patients were treated with GK for residual or recurrent GH-secreting adenoma. *Results*. Sixty-three patients (61.2%) reached the main outcome of the study. The rate of remission was 58.3% at 5 years (95% CI 47.6–69.0%). Other 15 patients (14.6%) were in remission after GK while on treatment with somatostatin analogues. No serious side effects occurred after GK. Eight patients (7.8%) experienced a new deficit of pituitary function. New cases of hypogonadism, hypothyroidism, and hypoadrenalism occurred in 4 of 77 patients (5.2%), 3 of 95 patients (3.2%), and 6 of 100 patients at risk (6.0%), respectively. *Conclusion*. In a highly selected group of acromegalic patients, GK treatment had good efficacy and safety.

## 1. Introduction

Acromegaly is an endocrine disorder that results from chronic secretion of abnormally high amounts of growth hormone. It is associated with increased morbidity and mortality; mortality is 2-3 times that of an age- and sex-matched normal population. Therapeutic options currently consist of surgical removal of the pituitary tumor, somatostatin analogs (SSA), GH receptor antagonists, dopamine agonists, and radiation therapy. The aims of therapy are to restore GH and IGF-I levels to normal and control tumor growth. Transsphenoidal surgery is the treatment of choice in the majority of acromegalic patients [1–5]. However, even in experienced hands, surgery leads to remission of acromegaly in about 60% of patients [6–8]. Those not cured by surgery or who have late recurrence of disease need other treatments, such as drugs [9, 10] or radiation [11, 12].

Single-session radiosurgery with the Leksell Gamma Knife (GK) permits to deliver high-dose radiation to a targeted volume. The surrounding normal structures are spared because of the steep fall-off of radiation at the margins of the lesion. Landolt et al. [13] showed that the highly precise and potent radiation delivered by GK caused a more rapid fall of GH levels than fractionated radiotherapy. However, there are few published data about the long-term results in acromegalic patients of radiosurgical treatment, including our previous study on a smaller group of patients with shorter follow-up [14].

The aim of our study was to evaluate the efficacy and safety of GK in a homogeneous cohort of acromegalic patients who had previously undergone maximal surgical debulking.

## 2. Clinical Material and Methods

### 2.1. Patient Population

We included in the study 112 consecutive patients who were treated with GK for residual or recurrent acromegaly, between January 1994 and December 2010. Diagnosis of active acromegaly was based on the clinical picture, GH levels not suppressed less than 1 ng/mL after a glucose load, and an elevated age- and sex-adjusted IGF-I level. Moreover, magnetic resonance imaging (MRI) showed a residual or recurrent pituitary tumor in each patient. The mean age at treatment was 42.2 ± 1.1 years (range 22–67 years). There were 68 women (60.7%) and 44 men (39.3%). Ninety-one patients (81.2%) had undergone surgery once, nineteen (17.0%) twice, and two patients (1.8%) four times. Hyperprolactinemia was detected in six patients (5.4%). Neuro-ophthalmological examination was normal in hundred eight patients (96.4%) and abnormal in four (3.6%) (Table 1).

 From 1994 to 2000, concomitant therapy with SSA was permitted according to patient's preference. After the release of one study that suggested a radioprotective effect of SSA [15], we advised, when feasible, to quit such treatment before GK. Because of this policy, only 20 patients (17.9%) were receiving SSA at the time of GK. Medical treatment was not initiated (66 patients) or was discontinued (36 patients) at least 4 months or 2 weeks before GK, depending on the formulation of the drug. Eleven patients (9.8%) continued taking dopamine agonists until the treatment with GK was performed. Standard informed consent was obtained from each patient undergoing GK. No patient was taking the GH-antagonist Pegvisomant.

### 2.2. Clinical and Hormonal Evaluation

Evaluation of pituitary function included measurement of free urinary cortisol excretion and basal serum GH, IGF-I, free T_3_, free T_4_, TSH, LH, FSH, PRL, cortisol, testosterone (in men), and 17-beta-estradiol levels (in premenopausal women). Neuroophthalmological examination included visual acuity testing, oculomotor function, and automated perimetry. During follow-up, GH and IGF-I levels determined without any concomitant medical therapy or after at least 4 months of discontinuation of SSA were considered as off treatment. Scheduled follow-up studies included MRI and neuroophthalmological examination 6, 12, 24, 36, and 48 months after GK and then at 2-year intervals, whereas GH, IGF-I, and pituitary function were determined at 6-month intervals for the first 2 years and then yearly thereafter or when clinically indicated.

All examinations were performed at the Istituto Scientifico San Raffaele whenever possible. Otherwise, the referring physicians performed testing at a local facility, and the results were sent to us for review. During the study period, a variety of assays were used to determine IGF-I levels. When indicated, we converted IGF-I levels into the multiple of the upper normal limit (mUNL).

The criterion for remission of acromegaly after GK was the achievement of normal age- and sex-adjusted IGF-I in combination with a GH level less than 2.5 ng/mL without concomitant treatment with GH-suppressive drugs. Patients who still needed GH-suppressive drugs were not considered in remission, irrespective of IGF-I and GH levels. Hypogonadotropic hypogonadism was diagnosed in premenopausal women with amenorrhea and in men with subnormal testosterone levels. Low or normal gonadotropin levels were also required in both sexes. Hypogonadism was also assigned to postmenopausal women with inappropriately normal gonadotropin levels; three premenopausal women taking estrogens were excluded from the analysis of gonadal function. Secondary hypothyroidism was diagnosed in patients with low free T_4_ level and normal or suppressed TSH concentration; four patients, already on T_4_ replacement therapy because of other thyroid disorders, were excluded from this analysis. Secondary hypoadrenalism was diagnosed by low 24 h free urinary cortisol and morning serum cortisol (<50 ng/mL) levels. Moreover, patients with serum cortisol levels in the lower half of the normal range were also started on a replacement therapy if they had symptoms of hypoadrenalism.

### 2.3. Gamma Knife Radiosurgery

A Leksell stereotactic head frame (model G; Elekta Instruments, Stockholm, Sweden) was positioned under mild sedation and after application of a local anesthetic agent. Magnetic resonance images (Siemens, Magneton Vision, 1,5 Tesla, Erlangen, Germany; Philips, Aciva, 1,5 Tesla, Eindhoven, The Netherlands) were performed for tumor visualization. The MRI sequences were: T_1_-weighted and T_2_-weighted without contrast and T_1_-weighted with contrast; slices were performed every 2 mm on three planes. Treatment was then planned with the KULA dose-planning software until 1995 and the Leksell GammaPlan system (Elekta Instruments) thereafter. GK was performed using a 201-source 60Co gamma knife (model B until December 2001 and model C thereafter). A neurosurgeon delineated the target and one radiotherapist approved the definitive radiosurgical planning. The entire residual tumor was covered within the 50% isodose line. The mean tumor volume was 1.8 ± 0.2 cc (range 0.1–7.2 cc). The goal of treatment was to deliver 25 Gy to the margin of the tumor. The mean prescription dose was 22.5 ± 0.3 Gy (range 12–25 Gy). Multiple isocenters were distributed throughout the target volume to conform the dose to the tumor margins. To this aim, small collimator sizes (4 and 8 mm) were used, and frequent source blocking was applied to obtain a sharper dose decrease toward the optic nerves, chiasm, and pituitary stalk. The dose to the tumor was decreased, when necessary, to keep a maximal dose of 10 Gy to the optical pathway. All patients were discharged the day after GK treatment.

### 2.4. Statistical Analysis

Continuous variables were examined for homogeneity of variance by the Kolmogorov-Smirnov test. For continuous variables with a normal distribution, the mean (SEM) is reported. For variables not normally distributed, the median and interquartile ranges (IQRs) are reported. The Wilkoxon signed-rank test for paired data was used to compare GH and IGF-I levels before and after GKR. Estimates of the cumulative event rate were calculated by the Kaplan-Meier method, and differences in subgroups of patients were tested by the log-rank test. Data for patients who were lost to follow up or who did not reach remission of disease were censored at the time of the last hormonal evaluation. Adjusted analysis of the primary outcome, that is, remission of acromegaly, was performed with the use of a Cox proportional-hazards regression model with the factors that had a *P* < 0.10 in the univariate analysis plus preidentified covariates of interest. A probability value of less than 0.05 was considered to indicate statistical significance, and all reported values are two sided. All calculations were performed using a commercially available statistical software package (SPSS 11.0 for Mac OS X; SPSS Inc., Chicago, IL).

## 3. Results

### 3.1. Long-Term Effects of GK on GH and IGF-I Levels

The median follow-up was 75 months (IQR 38–111 months; range 6–192 months). Seventy-nine patients (70.5%) did not receive any medical treatment at their last follow-up visit. Their median serum GH level fell from a baseline value of 5.1 ng/mL (IQR 2.9–10.0) to a one of 1.0 ng/mL (IQR 0.6–2.1; *P* < 0.001). Similarly, the median IGF-I level fell from 500 ng/mL (IQR 400–728) to 208 ng/mL (IQR 155–288; *P* < 0.001) (Table 2).

Remission of disease occurred in 68 patients (60.7%). Survival analysis showed that the probability to achieve remission of acromegaly was 30.7% at 3 years (95% confidence interval (CI) 21.5–39.9%) and 56.9% at 5 years (95% CI 46.5–67.3%). Further cases of remission occurred during prolonged follow-up so that the estimated 10-year rate of remission was 80.4% (95% CI 68.2–92.6%). Only two patients had recurrences of acromegaly 1 and 10 years after remission. The first patient was successfully treated with SSA, while the second patient, who was resistant to somatostatin analogues, received a second course of GK treatment. No other recurrence occurred in the other 61 patients. Other 18 patients (16.1%) had remission of disease while continuing SSA. Three patients normalized IGF-I levels after starting therapy with a GH receptor antagonist 3–6 yr after GK. In our study we did not find out any correlation between treatment volume and failure or remission rate.

### 3.2. Tumor Growth Control

At the last follow-up, tumor size remained unchanged in 61 patients (54.5%), decreased in 48 patients (42.9%), and increased in the remaining 3 patients (2.7%) (Table 3).

 Growth of the tumor occurred 4, 5, and 10 years after GK, respectively. However, one of the patients did not show recurrence of GH hypersecretion. In all three cases, recurrence occurred in an area that was not covered by GK because MRI at the time of treatment did not show any pathological tissue in that location. Two patients were treated with SSA and the third by a second GK. In all cases, there was no further tumor growth.

### 3.3. Side Effects of GK

No serious side effects occurred after GK. Ten patients (9.7%), three of whom already symptomatic before GK, complained of severe headache for at least 1 month after GK. No patient had deterioration of visual function or oculomotor function. One patient had CSF rhinoliquorrhea necessitating surgical repair 18 months after GK. The patient underwent transsphenoidal surgery with autologous fat apposition to seal the leak. Eight of 102 patients (7.8%) experienced a new deficit of pituitary function (the remaining patient had hypopituitarism before GK). In more detail, new cases of hypogonadism occurred in four of the 82 patients at risk (4.9%). New cases of hypothyroidism occurred in four of the 103 patients at risk (3.9%), and new cases of hypoadrenalism occurred in six (5.5%) of the 109 patients at risk. In all cases, replacement therapy was initiated accordingly. No cases of diabetes insipidus occurred after GK.

## 4. Discussion

Radiotherapy has the potential to obtain definitive remission of acromegaly, but the disadvantages of radiation include slow-onset effect on GH secretion, high risk of hypopituitarism, and rare but severe side effects, such as radionecrosis and secondary brain tumors [16, 17]. However, most information on the positive and negative effects of radiotherapy pertains to fractionated radiotherapy [11, 12, 18, 19]. GK has the advantage of delivering a highly focused radiation in a single fraction to the target lesion. This should lead to a faster decline of GH and IGF-I levels and a lower risk of complications. If such promises can be demonstrated in a sufficient number of patients, the indications for GK might be broadened in the future [1]. 

Our data, in a selected population of patients, show that remission occurred throughout the follow-up period, approaching almost 85% 10 yr after GK. Moreover, 18 patients, who had complete or partial resistance to SSA, achieved remission of disease while continuing the drug after GK. Recurrence of disease once remission had been achieved was quite uncommon.

Previous studies reported mixed results [20–25]. Using biochemical criteria similar to ours, remission of acromegaly occurred at 5 yr in 56–60% of cases [23, 24], whereas lower rates (29–30%) have been reported by other authors [20, 21]. Minor differences in the criteria of remission are unlikely to explain these differences because normalization of age- and sex-adjusted IGF-I levels was common to all these series. We, as other authors, did not require suppression of GH levels after oral glucose tolerance test as a criterion of remission because radiation therapy may alter GH feedback regulation, thus making the interpretation of GH dynamic testing more difficult [26]. Indeed, Powell and coworkers [27] showed a clear overlap in the postglucose GH levels in 15 irradiated patients, whose disease status was defined by IGF-I levels. GH and IGF-I levels before GK treatment and in the absence of concomitant GH-suppressive therapy were inversely associated with remission. The other variables, including sex, age, year of GK, concomitant treatment with SSA, and radiation dose to the tumor margin, were not independently associated with outcome. In a univariate analysis, basal GH and IGF-I levels off medication were inversely related to a successful outcome in two series [21, 23], whereas Pollock et al. [24], who also used a multivariate analysis, found that only baseline IGF-I levels had an independent predictive value. Interestingly, the same relationship between baseline hormone levels and remission of acromegaly also exists after conventional radiotherapy [12, 28–30]. Despite some exceptions to this supposition [18, 20, 25, 31], it seems reasonable that the less hormonally active tumors will normalize earlier because the kinetic of GH reduction after radiation seems to be independent of the starting GH level [21]. Therefore, maximal surgical debulking before GK should enhance the subsequent probability of success. In keeping with another series, [24] we found that the prescription dose to the tumor was not independently associated with remission of disease. The role of concomitant therapy with SSA has been debated after the report of Landolt et al. [15], which showed a clearly reduced efficacy of GK in patients while on medication. The supposed radioprotective effect of SSA has been confirmed by some [24] but not all authors [20, 21]. Only a randomized prospective study would give the final answer to this question. In the meantime and in keeping with other authors' point of view [21, 32, 33], we prefer quitting SSA before GK treatment, when clinically feasible.

Reduction of tumor size after GK occurred in 48.5% of our patients, but it was not related to biochemical outcome, as already reported by Ježková et al. [23]. Three patients showed growth of residual tumor located outside the area initially covered by GK. We described two similar cases in patients with a nonfunctioning pituitary adenoma [34]. Continuous MRI follow-up is therefore necessary, especially in patients without remission of disease. Determining the safety of GK is of paramount importance to broaden its use in acromegaly. The conventional radiosurgical tolerance dose of the anterior visual pathways is considered to be 8 to 10 Gy, and it is assumed that there is no serious risk of visual deficit due to radiation if no more than 15 Gy are delivered [13, 35–37]. In our study we did not experience any visual disturbances or deficits after Gamma Knife maintaining the dose to the optic pathway up to 10 Gy.

No serious side effect attributable to GK has occurred in our series. A similar safety profile has been described by other authors [20, 23]. Only two series [21, 24] reported serious side effects in three patients. However, they all had received conventional radiotherapy before GK, suggesting that the cumulative exposure to radiation rather than GK itself was the principal risk factor for serious complications.

New deficit of pituitary function occurred rarely in our series. In one study with a mean follow-up of 54 months [23], new cases of hypogonadism, hypothyroidism, and hypoadrenalism occurred in 41.1, 31.7, and 14% of the patients at risk, respectively. The rather high rate of hypopituitarism in that study might be attributed to the high percentage of patients (12.5%) who had previously received conventional radiotherapy and to the higher median radiation dose to the tumor margin (35 versus 22.5 Gy). The latter factor is probably the most important because another series that used a median margin dose similar to ours (20 Gy) had 5.3, 0, and 7.7% new cases of hypogonadism, hypothyroidism, and hypoadrenalism, respectively [20]. Pollock et al. [24] reported that 13 of 39 patients (33%) suffered a new pituitary deficit after a median follow-up of 63 months.

A direct comparison between the results of GK and fractionated radiotherapy is difficult to perform because patients selected to undergo GK may have more favorable characteristics, that is, smaller tumor size and lower GH levels, than those receiving fractionated radiotherapy. A faster normalization of IGF-I levels has been found in patients treated by GK than in a group of historical controls treated at the same institution by fractionated radiotherapy [13]. The risk of new-onset hypopituitarism seems, on average, to be lower for GK than fractionated radiotherapy. Only prospective, randomized, controlled studies would clarify the issue but are unlikely to be performed.

## 5. Conclusion

In conclusion, our study wants to demonstrate that, in a highly selected group of acromegalic patients subjected to previous surgery at our center, GK was effective and safe. This may lead to reconsider the role of GK in the therapeutic algorithm of acromegaly. GK treatment might be considered as an alternative to lifelong treatment with SSA or GH receptor antagonists, particularly in patients with small tumor residue located away from the optic pathway and the residual normal pituitary gland.

## Figures and Tables

**Table 1:** General characteristics of the population.

Characteristic	
Sex	
Male, *n* (%)	44 (39.3%)
Female, *n* (%)	68 (60.7%)

Age at GK (yr)	
Mean (±SEM)	42.2 (±1.1)

Previous surgery	
Once, *n* (%)	91 (81.2%)
Twice, *n* (%)	19 (17.0%)
>Twice, *n* (%)	2 (1.8%)

Hyperprolactinemia	
*n* (%)	6 (5.4%)

Neuro-ophthalmological examination	
Normal, *n* (%)	108 (96.4%)
Abnormal, *n* (%)	4 (3.6%)

Follow-up (months)	
Median (range)	71 (6–184)

Tumor volume (cc)	
Mean ± SEM (range)	1.8 ± 0.2 (0.1–7.2)

Prescription dose (Gy)	
Mean ± SEM (range)	22.5 ± 0.3 (12–25)

**Table 2:** Long-term effects of GK on GH and IGF- I levels in patients without medical treatment (*N* = 79).

Basal GH level (ng/mL)	Median (IQR)
Before GK	5.1 (2.9–10.0)
After GK	1.0 (0.6–2.1)

Basal IGF-I level (ng/mL)	Median (IQR)

Before GK	500 (400–728)
After GK	208 (155–288)

**Table 3:** Tumor growth control after GK treatment.

Tumor volume after GK	*n* (%)
Unchanged	61 (54.5%)
Decreased	48 (42.9%)
Increased	3 (2.7%)

## Abbreviations

GH: Growth hormone
PRL: Prolactin
IGF-I: Insulin-like growth factor-I
GK: Gamma Knife
CI: Confidence interval
IQR: Interquartile range
MRI: Magnetic resonance imaging
mUNL: Multiple of the upper normal limit
SSA: Somatostatin analog.

## References

[1] (2001). Treatment guidelines for acromegaly. Report from a Scandinavian workshop: first Scandinavian Workshop on the Treatment of Acromegaly. *Growth Hormone and IGF Research*.

[2] Besser GM, Burman P, Daly AF (2005). Predictors and rates of treatment-resistant tumor growth in acromegaly. *European Journal of Endocrinology*.

[3] Colao A, Martino E, Cappabianca P (2006). First-line therapy of acromegaly: a statement of the A.L.I.C.E. (Acromegaly primary medical treatment Learning and Improvement with Continuous Medical Education) Study Group. *Journal of Endocrinological Investigation*.

[4] Cook DM, Ezzat S, Katznelson L (2004). American Association of Clinical Endocrinologists Medical Guidelines for Clinical Practice for the diagnosis and treatment of acromegaly. *Endocrine Practice*.

[5] Melmed S, Casanueva FF, Cavagnini F (2002). Consensus: guidelines for acromegaly management. *The Journal of Clinical Endocrinology & Metabolism*.

[6] Beauregard C, Truong U, Hardy J, Serri O (2003). Long-term outcome and mortality after transsphenoidal adenomectomy for acromegaly. *Clinical Endocrinology*.

[7] Losa M, Mortini P, Urbaz L, Ribotto P, Castrignanò T, Giovanelli M (2006). Presurgical treatment with somatostatin analogs in patients with acromegaly: effects on the remission and complication rates. *Journal of Neurosurgery*.

[8] Nomikos P, Buchfelder M, Fahlbusch R (2005). The outcome of surgery in 668 patients with acromegaly using current criteria of biochemical ‘cure’. *European Journal of Endocrinology*.

[9] Melmed S, Casanueva F, Cavagnini F (2005). Consensus statement: medical management of acromegaly. *European Journal of Endocrinology*.

[10] Trainer PJ, Drake WM, Katznelson L (2000). Treatment of acromegaly with the growth hormone-receptor antagonist pegvisomant. *The New England Journal of Medicine*.

[11] Barrande G, Pittino-Lungo M, Coste J (2000). Hormonal and metabolic effects of radiotherapy in acromegaly: long-term results in 128 patients followed in a single center. *The Journal of Clinical Endocrinology & Metabolism*.

[12] Jenkins PJ, Bates P, Carson MN, Stewart PM, Wass JAH (2006). Conventional pituitary irradiation is effective in lowering serum growth hormone and insulin-like growth factor-I in patients with acromegaly. *The Journal of Clinical Endocrinology & Metabolism*.

[13] Landolt AM, Haller D, Lomax N (1998). Stereotactic radiosurgery for recurrent surgically treated acromegaly: comparison with fractionated radiotherapy. *Journal of Neurosurgery*.

[14] Losa M, Gioia L, Picozzi P (2008). The role of stereotactic radiotherapy in patients with growth hormone-secreting pituitary adenoma. *The Journal of Clinical Endocrinology & Metabolism*.

[15] Landolt AM, Haller D, Lomax N (2000). Octreotide may act as a radioprotective agent in acromegaly. *The Journal of Clinical Endocrinology & Metabolism*.

[16] Brada M, Ford D, Ashley S (1992). Risk of second brain tumour after conservative surgery and radiotherapy for pituitary adenoma. *British Medical Journal*.

[17] Tsang RW, Laperriere NJ, Simpson WJ, Brierley J, Panzarella T, Smyth HS (1993). Glioma arising after radiation therapy for pituitary adenoma. A report of four patients and estimation of risk. *Cancer*.

[18] Biermasz NR, van Dulken H, Roelfsema F (2000). Postoperative radiotherapy in acromegaly is effective in reducing GH concentration to safe levels. *Clinical Endocrinology*.

[19] Swearingen B, Barker FG, Katznelson L (1998). Long-term mortality after transsphenoidal surgery and adjunctive therapy for acromegaly. *The Journal of Clinical Endocrinology & Metabolism*.

[20] Attanasio R, Epaminonda P, Motti E (2003). Gamma-knife radiosurgery in acromegaly: a 4-year follow-up study. *The Journal of Clinical Endocrinology & Metabolism*.

[21] Castinetti F, Taieb D, Kuhn JM (2005). Outcome of gamma knife radiosurgery in 82 patients with acromegaly: correlation with initial hypersecretion. *The Journal of Clinical Endocrinology & Metabolism*.

[22] Gutt B, Wowra B, Alexandrov R (2005). Gamma-knife surgery is effective in normalising plasma insulin-like growth factor I in patients with acromegaly. *Experimental and Clinical Endocrinology and Diabetes*.

[23] Ježková J, Marek J, Hána V (2006). Gamma knife radiosurgery for acromegaly—long-term experience. *Clinical Endocrinology*.

[24] Pollock BE, Jacob JT, Brown PD, Nippoldt TB (2007). Radiosurgery of growth hormone-producing pituitary adenomas: factors associated with biochemical remission. *Journal of Neurosurgery*.

[25] Vik-Mo EO, Oksnes M, Pedersen PH (2007). Gamma knife stereotactic radiosurgery for acromegaly. *European Journal of Endocrinology*.

[26] Peacey SR, Toogood AA, Shalet SM (1998). Hypothalamic dysfunction in “cured” acromegaly is treatment modality dependent. *The Journal of Clinical Endocrinology & Metabolism*.

[27] Powell JS, Wardlaw SL, Post KD, Freda PU (2000). Outcome of radiotherapy for acromegaly using normalization of insulin-like growth factor I to define cure. *The Journal of Clinical Endocrinology & Metabolism*.

[28] Epaminonda P, Porretti S, Cappiello V, Beck-Peccoz P, Faglia G, Arosio M (2001). Efficacy of radiotherapy in normalizing serum IGF-I, acid-labile subunit (ALS) and IGFBP-3 levels in acromegaly. *Clinical Endocrinology*.

[29] Gutt B, Hatzack C, Morrison K, Pöllinger B, Schopohl J (2001). Conventional pituitary irradiation is effective in normalising plasma IGF-I in patients with acromegaly. *European Journal of Endocrinology*.

[30] Littley MD, Shalet SM, Swindell R, Beardwell CG, Sutton ML (1990). Low-dose pituitary irradiation for acromegaly. *Clinical Endocrinology*.

[31] Cozzi R, Barausse M, Asnaghi D, Dallabonzana D, Lodrini S, Attanasio R (2001). Failure of radiotherapy in acromegaly. *European Journal of Endocrinology*.

[32] Jackson IMD, Noren G (1999). Role of gamma knife radiosurgery in acromegaly. *Pituitary*.

[33] Zhang N, Pan L, Wang EM, Dai JZ, Wang BJ, Cai PW (2000). Radiosurgery for growth hormone-producing pituitary adenomas. *Journal of Neurosurgery*.

[34] Losa M, Valle M, Mortini P (2004). Gamma knife surgery for treatment of residual nonfunctioning pituitary adenomas after surgical debulking. *Journal of Neurosurgery*.

[35] Kenai H, Yamashita M, Nakamura T, Asano T, Sainoh M, Nagatomi H (2005). Tolerance dose in gamma knife surgery of lesions extending to the anterior visual pathway. *Journal of Neurosurgery*.

[36] Leber KA, Berglöff J, Pendl G (1998). Dose-response tolerance of the visual pathways and cranial nerves of the cavernous sinus to stereotactic radiosurgery. *Journal of Neurosurgery*.

[37] Tishler RB, Loeffler JS, Lunsford LD (1993). Tolerance of cranial nerves of the cavernous sinus to radiosurgery. *International Journal of Radiation Oncology Biology Physics*.

